# Structural Analysis of the AlkB Family in Poultry

**DOI:** 10.3390/ani15131942

**Published:** 2025-07-01

**Authors:** Yuling Niu, Kan Li, Xuerong You, Yutao Wu, Xue Du, Ayong Zhao, Zhijun Wang

**Affiliations:** 1 College of Animal Science and Technology, Zhejiang Agriculture and Forestry University, Hangzhou 311300, China; nyl@stu.zafu.edu.cn (Y.N.); qwqlovvv@163.com (X.Y.); taozi_02_09@163.com (Y.W.); duxue@zafu.edu.cn (X.D.); zay503@zafu.edu.cn (A.Z.); 2 Department of Animal Genetics, Breeding and Reproduction, College of Animal Science, South China Agricultural University, Guangzhou 510642, China; lidashuaibi.1@gmail.com; 3 National-Local Joint Engineering Research Center for Livestock Breeding, Guangdong Provincial Key Lab of Agro-Animal Genomics and Molecular Breeding, Guangzhou 510642, China

**Keywords:** AlkB family, poultry, bioinformatics, gene expression, phylogenetics

## Abstract

The objective of this study was to identify the AlkB family genes in poultry using bioinformatics, and to explore their molecular characteristics, evolutionary relationships, and expression patterns to clarify their potential functions in poultry. (1) Methods: The study utilized the NCBI database to obtain chicken genome data, and screened and validated AlkB family members (ALKBH1-5, ALKBH8, and FTO) by hmmsearch and TBtools. MEGA 11.0 was used for phylogenetic analysis, PHYRE2 and I-TASSER predicted protein structures, and the String database was used to construct an interoperability network. Finally, the tissue expression profiles were analyzed by using The Human Protein Atlas online database and qRT-PCR. (2) Results: Phylogenetic analysis revealed distinct avian and mammalian clusters, with chicken AlkB proteins exhibiting low sequence homology but conserved 3D structures compared to mammals. Chromosomal synteny and conserved domains highlighted evolutionary divergence, with ALKBH4 lacking typical AlkB structural motifs. Protein interaction networks linked ALKBH1/2/3/5/8/FTO, underscoring functional coordination in poultry adaptation. Tissue-specific expression showed high AlkB levels in brain tissues, while ALKBH5 dominated in muscle. During differentiation, ALKBH3, ALKBH5, and FTO expression significantly increased during myoblast differentiation. (3) Conclusions: This study identified seven AlkB family genes in poultry, revealing their phylogenetic classification into two subfamilies, conserved structural domains, chromosomal synteny, and tissue-specific expression patterns.

## Simple Summary

The AlkB family proteins are Fe^2+^/2-oxoglutarate-dependent dioxygenases that catalyze oxidative demethylation of nucleic acids. The vertebrate AlkB family comprises nine members—ALKBH1, ALKBH2, ALKBH3, ALKBH4, ALKBH5, ALKBH6, ALKBH7, ALKBH8, and FTO. In this study, using computer-based analysis and lab experiments, we identified seven AlkB genes in poultry and compared their evolution, structure, and activity across tissues. These genes are organized uniquely in poultry compared to mammals. ALKBH5, for example, showed high activity in muscle and brain tissues and an increased trend during muscle growth, suggesting its role in development. ALKBH6 and ALKBH7, two AlkB family genes present in mammals but not poultry, may be attributed to the substrate preferences exhibited by AlkB family proteins in poultry. By uncovering these genetic mechanisms, this research supports the breeding of healthier, more resilient poultry, as controlling health and growth in poultry is vital for improving farming and disease resistance.

## 1. Introduction

Members of the AlkB family typically possess α-ketoglutarate (α-KG)- and Fe (II)-dependent dioxygenase activity, which may endow them with the potential to catalyze demethylation reactions involving various methylation modifications in both RNA and DNA [1]. The two currently reported m^6^A demethylases, ALKBH5 and FTO, both belong to the AlkB family. Earlier studies found that FTO could participate in the deoxy methylation of m^3^T [2], m^3^U [3], etc. However, in 2011, by synthesizing m^6^A-specific single-stranded DNA (ssDNA) and single-stranded RNA (ssRNA), researchers discovered for the first time that FTO exhibited demethylase activity against N6-methyladenine (m^6^A) in both DNA and RNA [4]. In 2013, researchers demonstrated the demethylase activity of ALKBH5 by constructing ALKBH5 knockout mice, which showed genetic modification-impaired spermatocyte apoptosis during meiosis, thus leading to reduced fertility [5]. Both ALKBH5 and FTO can participate in the reverse process of m^6^A methylation, thereby maintaining m^6^A methylation in dynamic equilibrium. ALKBH5 plays a crucial role in spermatogenesis during meiosis and haploid phases by removing m^6^A modifications to ensure the correct splicing and stability of longer 3′-untranslated region (3′-UTR) mRNAs [6]. In the context of RNA methylation applications, ALKBH5 also binds to human telomerase RNA (hTR), removes m^6^A modifications, and influences telomerase assembly and activity [7]. FTO, a fat-associated and obesity-associated protein, promotes adipogenesis, and its activity correlates with susceptibility to obesity. FTO’s involvement in the selective pre-mRNA splicing affects adipocyte differentiation in mice [8]. Additionally, FTO-mediated demethylation of m^6^A has also been shown to enhance cell viability and accelerate cell proliferation in acute myeloid leukemia cells, inhibit apoptosis in cancer cells, and increase the risk of infectious diseases [9]. Therefore, understanding the binding sites of FTO may offer insights into reducing cancer incidence to a certain extent. Research has found that ALKBH5 and FTO are differentially expressed in the livers of mice at various growth stages, suggesting their involvement in the regulation of liver development and maturation [10].

Beyond FTO and ALKBH5, other members of the AlkB family have been implicated in a broad spectrum of human diseases and physiological processes. For example, ALKBH1, which has been implicated in gastric cancer and glioblastoma, has the potential to induce mitochondrial damage by targeting N6-methyladenine (N6mA) in DNA and exerting its demethylase activity, thereby downregulating and disrupting genes encoded by mitochondrial DNA [11]. ALKBH2 and ALKBH3 function as DNA repair enzymes in vivo, and their overexpression may contribute to tumorigenesis in humans [12]. Specifically, ALKBH2 is a DNA repair enzyme that affects colorectal cancer (CRC) cell proliferation by regulating the B cell-specific Moloney murine leukemia virus integration site 1 (BMI1)-mediated NF-κB signaling pathway [13]. Meanwhile, ALKBH3 can catalyze the demethylation of N1-Methyladenosine (m^1^A) and 3-Methylcytidine (m^3^C) in both mRNA and tRNA [14,15]. In ovarian and breast cancers, ALKBH3 can methylate m^1^A in the 5′-Untranslated Region (5′-UTR) near the translational site, thus reducing the expression of CSF-1 mRNA in cancer cells [16]. Furthermore, ALKBH7 and ALKBH8 have been linked to prostate and bladder cancers, respectively. Notably, ALKBH7 was found to act as an RNA methylase and a naturally occurring N2, N2-dimethylguanosine (m_2_^2^G) demethylase in mammalian mitochondria. It mediates the unprecedented m_2_^2^G and m1A demethylation, thereby regulating polycistronic mitochondrial RNA (mtRNA) processing and mitochondrial function [17]. Leonardi et al. [18] constructed an ALKBH8 deletion model in mouse lung fibroblasts injured by naphthalene, showing increased tolerance to oxidative stress. They found that ALKBH8 regulates mitochondrial function via 5-methoxycarbonylmethyluracil (mcm^5^U) and 5-methoxycarbonylmethyluridine-2′-O-methyluridine (mcm^5^Um), which are involved in tRNA methylation, leading to higher selenoprotein levels, reduced oxidative stress tolerance, and alleviated lung injury. In summary, the AlkB family of enzymes, which comprises ALKBH1-8 and FTO, plays a significant role in RNA modification and DNA repair through oxidative demethylation. Emerging evidence underscores the diverse substrate specificities and gene-regulatory roles of AlkB family members, notably ALKBH5 and FTO, which possess RNA demethylase activity. Other family members may also exhibit RNA demethylase potential and be involved in human diseases and physiological processes.

Extensive research has been conducted on the demethylation of m6A by ALKBH5 and FTO across poultry and livestock. By suppressing FTO, the methylation of m6A can reduce the expression of heat stress protein (HSP) [19]. Notably, both FTO and ALKBH5 have been implicated in heat stress responses in sheep [20]. ALKBH5 can inhibit porcine alveolar epidemic diarrhea virus (PEDV) infection [21] and influence post-pubertal development in the bovine ovary [22]. In the field of poultry health and production, N6-Methyladenosine (m^6^A), an RNA modification associated with the AlkB family, is implicated in various biological processes, including muscle development [23], the growth of pre-fat and abdominal fat [24,25], and the metabolism of liver fat [26].

A study analyzing various bioinformatics databases like ONCOMINE, TIMER, and Disease-Meth revealed significant upregulation of ALKBH1, ALKBH2, ALKBH4, ALKBH5, ALKBH7, and ALKBH8 in lung adenocarcinoma (LUAD) tissues compared to normal lung tissues. Conversely, ALKBH3, ALKBH6, and FTO showed decreased expression in LUAD samples, hinting at epigenetic regulation in LUAD [27].

Despite the extensive research on the AlkB family predominantly focusing on model organisms like humans and mice, there is a possible lack of research on poultry. The specific roles and structural characteristics of the AlkB family in avian species remain elusive. Considering the significance of poultry in agriculture and livestock, as well as their crucial role as research models, studying the AlkB family could provide new avenues for their development. In this study, we employed bioinformatics tools to identify and characterize seven members of the AlkB family in poultry, including ALKBH1, ALKBH2, ALKBH3, ALKBH4, ALKBH5, ALKBH8, and FTO. Through a comprehensive analysis of their sequence structures, molecular characteristics, expression patterns, and evolutionary relationships across species, we gained valuable insights into their homology, structure, and expression patterns. These findings lay a theoretical foundation for potential therapeutic applications and genetic improvement in poultry.

## 2. Materials and Methods

### 2.1. Classification of Members of the AlkB Family

The FASTA sequence and annotation files for the chicken genome (GCF_016699485.2_bGalGal1.mat.broiler.GRCg7b_genomic.fna) were downloaded from the NCBI database. The coding sequence (CDS) of each gene was extracted from the chicken genome using the GTF/GFF3 sequence extractor module of the TBtools II v2.030 software [28]. The amino acid sequence files for the proteins were obtained through the “Batch Translate CDS to Protein” process of the TBtools software. To identify all potential AlkB family genes in chickens, all known Hidden Markov Model (HMMs) for the 2OG-Fe (II) oxygenase superfamily and the FTO catalytic domain of the AlkB family were obtained from the online website (https://pfam.xfam.org/ (accessed on 21 September 2024)), with PF03171 and PF12933 as the Pfam identifiers, respectively. Hmmsearch [29], with the default parameters (basic syntax: Hmmsearch FTO.hmm/2OG-Fe (II)_oxygenase_superfamily.hmm GRCg7b.protein.fa > results), was used to perform the preliminary screening of AlkB gene family members, ensuring the high quality of candidate genes (E-value < 1 × 10^−5^). After removing duplicate genes, the amino acid sequences of the candidate genes were used for further analysis and to retrieve the candidate genes of AlkB family members.

### 2.2. Phylogenetic and Structural Analysis of the Chicken AlkB Family

A phylogenetic tree was constructed using MEGA 11.0 software, employing the ClustalW and Neighbor-Joining methods, to further evaluate and screen the genes of the chicken AlkB family members. The amino acid sequences of all screened genes were downloaded from the UniPort database. Motif analysis and conserved domains were visualized using TBtools. The conserved domain information of the AlkB gene family was obtained from CD-research in NCBI.

### 2.3. Chromosomal Localization and Structural Prediction

The NCBI database was used to identify the relative genetic loci of the members of the AlkB gene family in different species, including humans, mice, chickens, ducks, and turkeys. The Phyre2 network server was used to predict the 3D structure of AlkB family proteins in humans and chickens, and these predictions were confirmed by I-Tasser [30,31].

### 2.4. Sequence Alignment and Phylogenetic Analysis of AlkB Family Proteins from Different Species

The amino acid sequences of AlkB family proteins from various species, including *Homo sapiens*, *Mus musculus*, *Sus scrofa*, *Bos taurus*, *Ovis aries*, *Oryctolagus cuniculus*, *Cavia porcellus*, *Erinaceus europaeus*, *Canis lupus*, *Equus caballus*, *Loxodonta Africana*, *Felis catus*, *Camelus ferus*, *Pan troglodytes*, *Gorilla gorilla*, *Ornithorhynchus anatinus*, *Xenopus tropicalis*, *Xenopus laevis*, *Gallus gallus*, *Coturnix japonica*, *Anas platyrhnchos*, *Strigops habroptila*, *Meleagris gallopavo*, *Columba livia*, *Taeniopygia guttata*, *Ficedula albicollis*, *Tyto alba*, *Egretta grazetta*, *Pavo cristatus*, *Danio rerio*, *Oreochromis niloticus*, *Salmo salar*, and *Esox Lucius*, were downloaded from the Uniprot database. The identities and differences in protein sequence within the AlkB family across different species were compared using SDT v1.3 software (http://web.cbio.uct.ac.za/SDT (accessed on 18 October 2024)). The Bootstrap and Neighbor-Joining methods were utilized to construct the phylogenetic tree in the MEGA software.

### 2.5. Experimental Animals and Cells

The 20-week-old Xinghua (XH) chicken (*N* = 6) tissues used in this study were previously collected from the Guangdong Provincial Key Lab of Agro-Animal Genomics and Molecular Breeding. Tissues from the heart, liver, spleen, lung, kidney, pectoral muscle, leg muscle, brain, cerebellum, hypothalamus, skin, abdominal fat, and subcutaneous fat were used for qRT-PCR analysis of the AlkB family’s gene expression profiles. Eggs laid 11 days prior were used to isolate chicken primary myoblasts. The chicken primary myoblast isolation protocol was as previously described [32].

### 2.6. RNA Extraction, cDNA Synthesis, and qRT-PCR

Total RNA extraction of tissues and cells was performed using the Trizol Reagent and chloroform isopropanol method. A total of 1 μg RNA per sample was used as the input material for reverse transcription (*N* = 6). MonScript™ RTIII All-in-One Mix with dsDNase Kit (Monad, Suzhou, China) was used for cDNA synthesis. The CDS sequences of the genes used in the experiments were downloaded from the NCBI database, and the specific quantitative real-time PCR (qRT-PCR) primers were subsequently designed using Oligo 7 software. The primer information is shown in Table 1. The qRT-PCR reactions were carried out using a CFX96 Real-Time PCR System (Bio-Rad, Hercules, CA, USA) with the AceQ Universal SYBR qPCR Master Mix (Vazyme, Nanjing, China) with three independent replications. The 2^−ΔΔct^ method was used for calculating the relative expression of mRNA [33].

## 3. Results

### 3.1. Screening and Identification of Chicken AlkB Family Members

**Figure 1:**
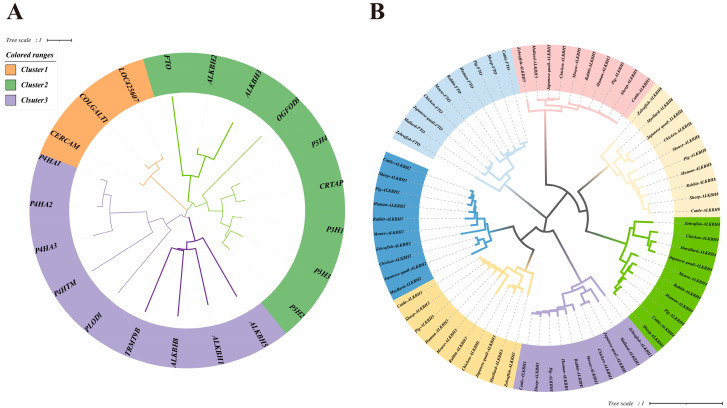
Phylogenetic trees of chicken AlkB family genes. (**A**) Evolutionary tree for classification of chicken AlkB family genes; (**B**) phylogenetic evolutionary tree of ALKB family among different species.

To comprehensively analyze the members of the chicken AlkB protein family, we employed a robust approach involving the Hidden Markov Model of the established AlkB family, coupled with the bidirectional blast alignment feature of TBtools. This strategy yielded a total of 21 protein sequences. Subsequently, we constructed a rootless evolutionary tree to categorize all genes and delineate the distinct subfamilies within these protein sequences (Figure 1A).

Figure 1 reveals that LOC425607, COLGALT1, and CERCAM coalesce into Cluster 1, which contains only glycosyltransferase 25 family members. Conversely, Cluster 2 encompasses FTO, ALKBH2, ALKBH3, OGFOD3, P3H1, P3H2, P3H3, P3H4, and CRTAP, where P3H1, P3H2, P3H3, P3H4, and CRTAP belong to the leprecan family. Additionally, Cluster 3 groups P4HA1, P4HA2, P4HA3, P4HTM, PLOD1, TRMT9B, ALKBH1, ALKBH5, and ALKBH8, with P4HA1, P4HA2, and P4HA3 belonging to the P4HA family.

Focusing specifically on our objective to analyze the AlkB family in chickens, we excluded non-AlkB homologous proteins. Furthermore, TRMT9B, annotated as a putative gene in the NCBI database, was omitted from subsequent analyses. Consequently, ALKBH1, ALKBH2, ALKBH3, ALKBH5, ALKBH8, and FTO were selected as candidate genes for the chicken AlkB family. Intriguingly, within Cluster 2, FTO, ALKBH2, and ALKBH3 formed a subbranch, while ALKBH1, ALKBH5, and ALKBH8 constituted another subbranch in Cluster 3. Figure 1B displays a colorful dendrogram illustrating the distribution of AlkB family genes across various species, thereby revealing their evolutionary relationships. The results show that the phylogenetic tree structures of the family genes are basically similar except for ALKBH2 and ALKBH4.

### 3.2. Phylogenetic and Gene Structural Analysis of Chicken AlkB Family Members

**Figure 2:**
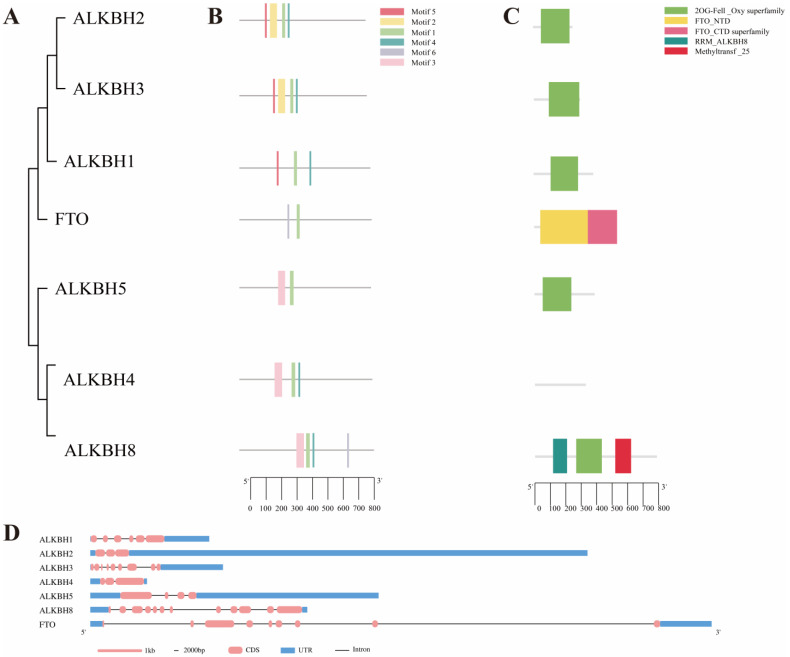
Phylogenetic tree, conserved motif, and gene structure of chicken AlkB family. (**A**) Classification chart of two main subfamilies of AlkB family; (**B**) AlkB family conserved structural domain motif analysis; (**C**) analysis of gene-specific structure of chicken AlkB family; (**D**) structural analysis of CDS sequence of genes in chicken AlkB family.

To investigate the evolutionary relationships among the genes belonging to the chicken AlkB family, a phylogenetic tree was constructed utilizing the meticulously screened protein sequences of its family members. Notably, ALKBH4, annotated as AlkB homolog 4 and lysine demethylase in the NCBI database, was purposefully incorporated into the analysis despite its absence in previous family gene screening. The phylogenetic tree revealed a dichotomous classification of the chicken AlkB family into two primary subfamilies (Figure 2A). One subfamily comprises the following four members: ALKBH1, ALKBH2, ALKBH3, and FTO. The other subfamily includes the following three members: ALKBH4, ALKBH5, and ALKBH8.

An analysis of the conserved motifs within the AlkB family, based on their evolutionary relationships, revealed a total of six conserved domains designated as motif 1, 2, 3, 4, 5, and 6 (Figure 2B). Motif 1 is common to all family members. Specifically, ALKBH1, ALKBH2, and ALKBH3 share motif 1, 4, and 5, whereas ALKBH4, ALKBH5, and ALKBH8 share motif 1 and 3 (Figure 2A,B). With the exception of FTO and ALKBH4, all other AlkB family proteins possess the 2OG-Fell_Oxy superfamily domain (Figure 2A,C). Using the genome annotation file, the gene CDS, untranslated regions (UTRs), and introns of the chicken AlkB family genes were visualized (Figure 2D). Among them, FTO exhibits the longest gene sequence length. Based on the aforementioned analysis, it appears that FTO lacks the 2OG-Fell_Oxy superfamily domain, which is present in other AlkB family members. Furthermore, the conservative domains FTO_NTD and FTO_CTD are distinctly different from those of other genes within the family.

### 3.3. Chromosomal Position Distribution and Collinearity Analysis of the AlkB Family Across Species

**Figure 3:**
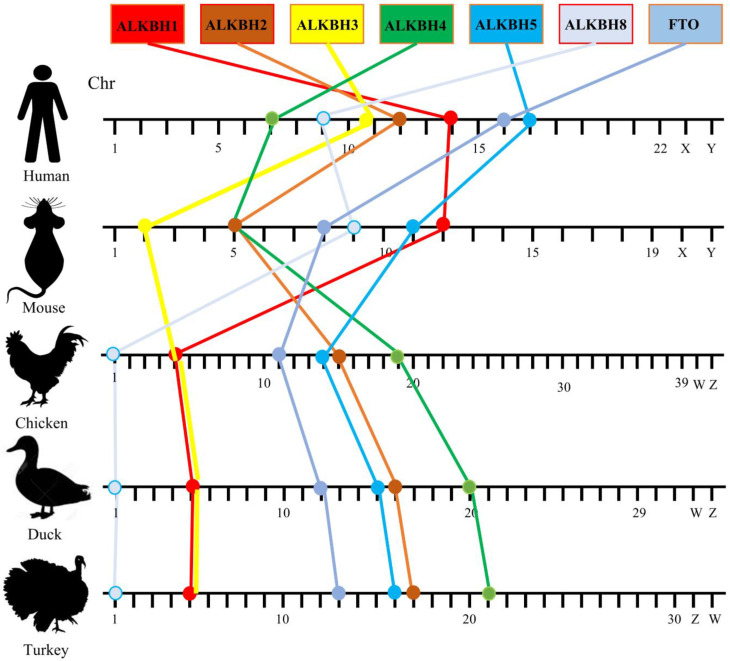
Chromosomal distribution and synteny comparison of AlkB family genes in humans, mice, chickens, ducks, and turkeys.

To precisely assess the chromosomal collinearity patterns of AlkB family genes in diverse species, we selected and compared closely related species, namely humans and mice (whose common ancestors date back approximately 40–80 million years), with avian species such as chickens, ducks, and turkeys (which diverged from mammals roughly 310 million years ago). The findings (Figure 3) revealed that all AlkB family genes in the chosen species are situated on autosomes, with a conserved loss of collinearity observed among the AlkB family genes in mice and humans. In stark contrast to mammals, the relative position and order of the poultry AlkB family genes (ALKBH1, ALKBH3, ALKBH8) on chromosomes 5 and 1 remain consistent in chickens, ducks, and turkeys. Meanwhile, the other four genes (ALKBH2, ALKBH4, ALKBH5, and FTO) exhibit similar chromosomal distribution patterns (arranged in a progressive sequence) across chickens, ducks, and turkeys (Figure 3). This underscores the robust collinearity of the AlkB family genes among these three avian species.

### 3.4. Phylogenetic Analysis of AlkB Family Proteins Across Species

**Figure 4:**
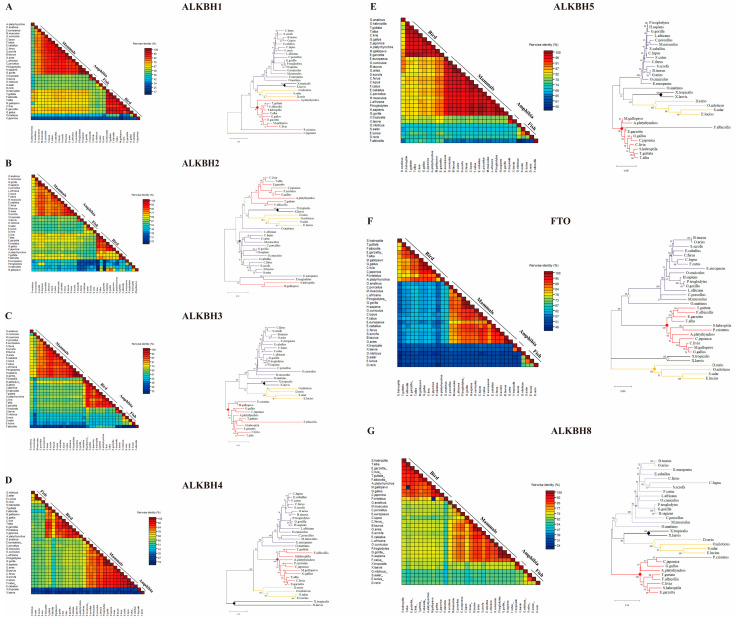
Precent identity comparison and phylogenetic analysis of AlkB family. Percentage of sequence identity and variation in AlkB protein sequence identity across species and phylogenetic trees of ALKBH1 (**A**), ALKBH2 (**B**), ALKBH3 (**C**), ALKBH4 (**D**), ALKBH5 (**E**), FTO (**F**), and ALKBH8 (**G**).

The homology percentages and variations in AlkB protein sequences among diverse species were meticulously examined using SDT programs (employing the Neighbor-Joining method). The MEGA software was used here to construct phylogenetic trees of the AlkB family. The results show that FTO protein sequences possess the lowest homology (55–64%) among AlkB family members in birds compared to mammals, while ALKBH5 possesses the highest homology (74–82%) (Figure 4E,F), and the rest of the percentages are in the descending order of ALKBH1, ALKBH8, ALKBH 2, ALKBH4, and ALKBH3 (Figure 4A–D,G). The phylogenetic trees reveal that AlkB family proteins can be clearly categorized into avian and mammalian clusters. In the evolutionary tree of ALKBH1, it was found that amphibians, fish, and mammals share the same evolutionary branch, unlike birds, which have a distinct evolutionary branch. ALKBH3, ALKBH5, and ALKBH8 have similar evolutionary patterns, whereas ALKBH2 has the opposite evolutionary relationship. Birds, fish, and amphibians belong to the same evolutionary branch, while mammals are clearly separated from them. Interestingly, birds and mammals belong to the same evolutionary branch in ALKBH4 and FTO relative to fish and amphibians. However, there is no cross-dispersion, i.e., birds and mammals still have clearly different evolutionary directions. In the phylogenetic bird clusters, it was found that ducks show higher variance in these genes compared to chickens and turkeys. In addition, both amphibians and fish have distinct evolutionary directions in these phylogenetic relationships.

### 3.5. Protein Characteristics, Structural Insights, and Protein–Protein Interaction Patterns of Chicken AlkB Family Genes

**Figure 5:**
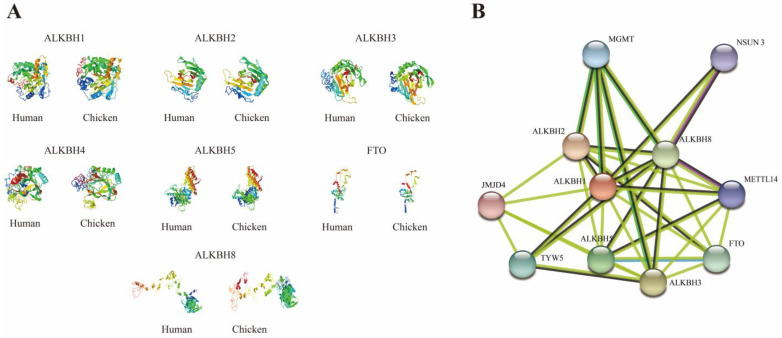
Protein structure prediction and interaction patterns of AlkB family. (**A**) 3D protein structure analysis of human and chicken AlkB family genes; (**B**) patterns of AlkB family protein interaction network.

Table 2 details the protein IDs, amino acid counts, molecular weights, and isoelectric points (pI) of the chicken AlkB family genes. Among the seven proteins, ALKBH2 stands out with the smallest molecular weight, comprising 247 amino acids, whereas ALKBH8 boasts the largest, encompassing 746 amino acids. The isoelectric points range from 5.74 (FTO) to 9.24 (ALKBH2), primarily dictated by the disparity in the counts of acidic and basic amino acids (a protein is deemed basic if its pI is > 7.0 and acidic if the pI is < 7.0). Following this, the 3D structures of human and chicken AlkB family proteins were analyzed and predicted by utilizing the I-Tasser online web server (Figure 5A); the interaction network of chicken AlkB family proteins was thoroughly analyzed by leveraging the online tool String (Figure 5B). The results unveiled a striking similarity in the 3D structures of these proteins, albeit with certain positional substitutions noted. Figure 5B suggests potential interactions among ALKBH1, ALKBH2, ALKBH3, ALKBH5, ALKBH8, and FTO. It is noteworthy that ALKBH4, which lacks protein structural characterization, did not demonstrate any identified interactions in this analysis.

### 3.6. The Expression Profiles of the AlkB Family Genes

**Figure 6:**
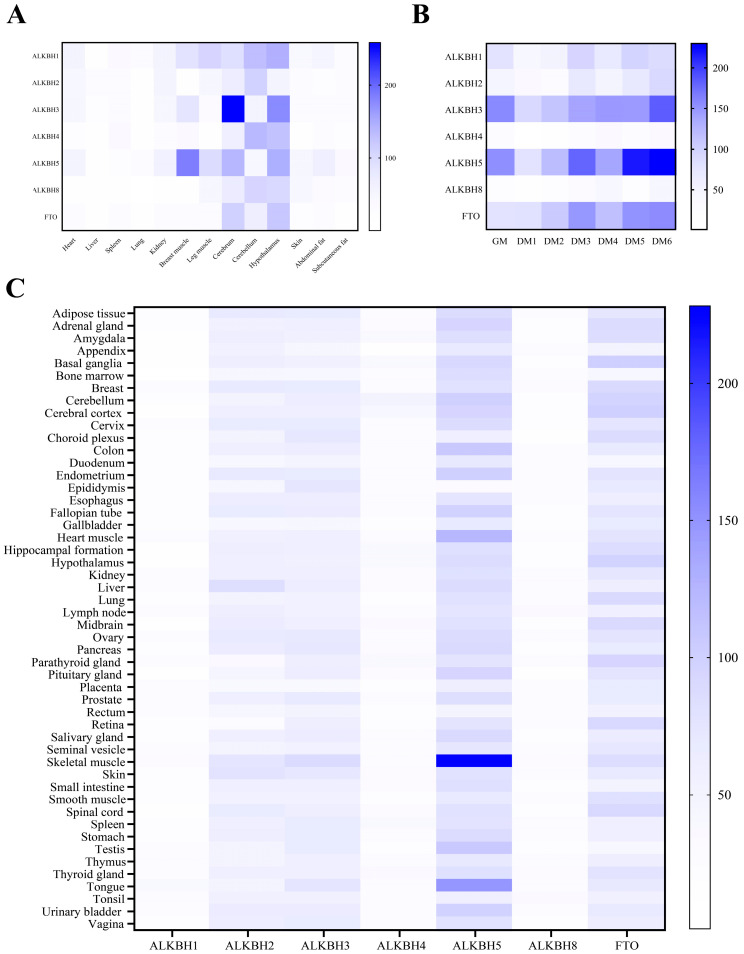
Gene expression status of AlkB family. (**A**) Tissue expression profiles of chicken AlkB family genes, determined by qRT-PCR in heart, liver, spleen, lung, kidney, breast muscle, leg muscle, cerebrum, cerebellum, hypothalamus, skin, abdominal fat, subcutaneous fat. (**B**) Time expression profiles of AlkB family genes during myogenic differentiation of chicken myoblast, determined by qRT-PCR. GM, growth medium, here means cell is in proliferation phase. DM, differentiation medium. First 24 h spent inducing myoblast differentiation was defined as day 1 (DM 1), and so on until day 6 (DM 6). (**C**) Tissue expression profiles of human AlkB family genes analyzed using The Human Protein Atlas.

Understanding gene expression patterns offers profound insights into gene functionality. To delve into the expression profiles of AlkB family proteins across various chicken tissues, we conducted a qPCR analysis of diverse AlkB genes in thirteen tissues (Figure 6A). Overall, AlkB family proteins were highly expressed in brain tissues (cerebrum, cerebellum, and hypothalamus). Among them, ALKBH3, ALKBH5, and FTO were highly expressed in the cerebrum and hypothalamus, ALKBH3 had the highest expression level in the cerebrum, and ALKBH2, ALKBH4, and ALKBH8 had the highest expression level in the cerebellum. It is worth noting that ALKBH5 exhibited its highest expression in breast muscle tissue. By analyzing the expression profiles of AlkB family genes across different human tissues using The Human Protein Atlas online database (The Human Protein Atlas), it was also found that ALKBH5 exhibited the highest expression level in muscle tissue, such as heart muscle, skeletal muscle, and the tongue (Figure 6C). This suggests a closer homology of ALKBH5 between avian animals and mammals. We also found that ALKBH5 had high expression levels in human testis, which also suggests a function of ALKBH5 in spermatogenesis. Furthermore, we investigated the expression dynamics of AlkB family genes during the differentiation of chicken primary myoblasts. Our findings revealed that the expression of AKLBH3, ALKBH5, and FTO showed a significant upregulation trend with the extension of differentiation time, while ALKBH4 and ALKBH8 showed almost no change in expression with the extension of time (Figure 6B).

## 4. Discussion

AlkB proteins execute oxidative dealkylation to eliminate alkyl adducts from nucleobases, with a prototypical and related member being the “adaptive response” protein in Escherichia coli that safeguards the bacterial genome against alkylation-induced damage [34]. Researchers have found that AlkB family genes are reliant on demethylation functions and play pivotal roles in diverse biological processes, such as RNA metabolism, DNA damage, and fatty acid metabolism [35]. Studies on ruminants fed low-protein diets supplemented with methionine and lysine have demonstrated an increase in m^6^A RNA methylation in the liver and muscle, accompanied by downregulation of ALKBH5 and FTO demethylase. These findings highlight FTO’s influence on fat metabolism [36]. Furthermore, dietary additions of betaine and cycloleucine have revealed that m^6^A levels could regulate fat accumulation in porcine adipocytes through FTO [37]. In human cellular research, the AlkB family has been extensively utilized for cancer management, with notable studies focusing on glioblastoma [38], ovarian cancer [39], hepatocellular carcinoma [1], breast cancer [40], and lung adenocarcinoma [27].

To gain insights into the chicken AlkB family, we constructed a phylogenetic tree by initially screening seven chicken AlkB family proteins, ALKBH1, ALKBH2, ALKBH3, ALKBH4, ALKBH5, ALKBH8, and FTO, based on the known structures of mammalian AlkB family proteins (Figure 1). ALKBH6 stands out among other AlkB family members due to its unique Flip1 and Flip2 structural domains, which differentiate it both in sequence and conformation [41]. ALKBH7 is postulated to be the enzyme responsible for direct demethylation in mammalian mitochondria [42]. Unlike mammals, we did not identify ALKBH6 and ALKBH7 in chickens, which may be attributed to the substrate preferences exhibited by the AlkB family proteins in poultry. We found that ALKBH4 does not belong to the AlkB family when we constructed the AlkB family phylogenetic evolutionary tree. In our study, we found that ALKBH4 does not have the 2OG-Fell_Oxy superfamily structural domain (Figure 2), and this structural difference may have led to the functional difference.

The ALKBH1 gene encodes a DNA demethylase primarily targeting N6-methyladenine (N6mA) in DNA, and it also functions as a tRNA demethylase, removing N1-methyladenine (m^1^A) from various tRNAs [43]. ALKBH2 demonstrates a preference for double-stranded DNA (dsDNA) as its substrate, detectable through its direct oxidative demethylation of methyl groups on ssDNA bases to repair alkylated nucleic acids, while also exhibiting activity towards dsDNA [44]. During evolution, ALKBH2 and ALKBH3 share a common phylogenetic branch, suggesting a shared ancestral origin and conserved structural domains with subtle differences, such as a double-stranded β-helix (DSBH) [45]. These structural nuances contribute to their progressive functional divergence, resulting in distinct substrate preferences [46]. Additionally, their distinct subcellular localizations may also influence functional divergence. Specifically, ALKBH2’s structure fits into double-stranded DNA grooves, stabilizing the double helix and facilitating catalysis, while ALKBH3’s simple structure with shallow grooves favors binding to single-stranded nucleic acids like mRNA [43]. ALKBH4 functions as a demethylase of actinomyosin K84me1, playing a crucial role in actin mitosis and meiosis [44]. ALKBH8 possesses an NRL structural domain and a DBSH structural domain in addition to the N-terminal and C-terminal RNA-recognition motifs (RRMs), respectively, contributing to its preference for tRNA as a substrate [17]. Notably, ALKBH8 also includes an RRM domain at the N-terminal end and a methyltransferase (MTase) domain at the C-terminal end. ALKBH5 and FTO are the demethylases within the AlkB family, often referred to as the m^6^A “erasers” of epigenetic information. Their demethylation enzyme catalytic activity is contingent upon the presence of the AlkB family signature, with catalytic activity reliant on Fe^2+^ and α-ketoglutarate (α-KG) [34]. Xu and colleagues [46] introduced the concept of a critical loop to elucidate the substrate specificity of the AlkB family. Due to these specialized structures, ALKBH5 and FTO differ in the mechanisms that mediate demethylation at the DNA level and at the RNA level. Specifically, the critical loops of the ALKBH5 and FTO form a narrower cleft, while FTO stands out among its eight family members for its unique non-homologous structure in the key loop. These special structures have significant implications for the substrate specificity of AlkB family proteins. In mammals, demethylation of 5-methylcytosine (5mC) at the DNA level primarily occurs through the iterative oxidation of 5mC to 5-hydroxymethylcytosine (5hmC), 5-formylcytosine (5fC), and 5-hydroxycytosine (5caC), whereas at the RNA level, the human AlkB homologues FTO and ALKBH5 are involved in the removal of m^6^A methylation through oxidation [47].

Intriguingly, our findings reveal that, compared to humans, avian species exhibit a loss of homozygosity in their AlkB family genes, yet these genes display remarkably similar protein structural patterns (Figure 5), which may be attributed to chromosomal rearrangements during species evolution. However, our study found that AlkB family genes are more strongly covaried in avian species (Figure 4). This strong covariance suggests that the AlkB family genes may be subject to more stringent evolutionary constraints in avian species, or they may be involved in a specific biological process that is conserved in avian species. In addition, this covariance may also reflect the relatively few chromosomal rearrangement events that have occurred in avian species during the course of evolution, thus maintaining the original arrangement of these genes on the chromosomes.

Through qPCR analysis of AlkB genes in avian pectoral muscles, we found that ALKBH5 expression was elevated in both human and chicken muscle tissues, while FTO lacked tissue specificity in humans but showed higher expression in chicken brain tissues (Figure 6). We analyzed the genotypes and gene expression levels of the AlkB family in chicken muscle tissue using an online database (http://chicken.farmgtex.org/ (accessed on 14 June 2025)). The data were consistent with our research findings (Figure 6), suggesting that FTO and ALKBH5 are promising candidate genes for regulating poultry muscle development and trait selection. Quantitative trait locus (QTL) analysis revealed that ALKBH5 is closely associated with muscle-related QTLs. In contrast, ALKBH1 and ALKBH4 lacked significant expression QTL (eQTL) signals associated with traits in muscle tissue, potentially indicating more conserved, non-specific functions. Significant differences in exon usage QTLs (exQTLs) were observed for ALKBH2, ALKBH3, ALKBH8, and FTO, which might reflect functional differentiation among family members during evolution. Furthermore, ALKBH1 and ALKBH3 exhibited more significant splicing QTLs (sQTLs) in muscle tissue. These genes may regulate muscle development and metabolism through mechanisms such as DNA repair, protein interactions, or other epigenetic modifications, thereby contributing to genetic diversity.

Corresponding studies also indicate that ALKBH5 undergoes demethylation in diverse chicken tissues [48,49]. M^6^A may be involved in the proliferation of bovine myoblasts and regulate skeletal muscle growth, as shown by Yang [16], where FTO knockdown promotes the proliferation of myoblasts to inhibit differentiation, while ALKBH5 knockdown inhibits cell proliferation, leading to apoptosis. A KEGG pathway analysis showed that ALKBH5 may be involved in “metabolic pathways”, “muscle contraction”, “actin cytoskeleton regulation”, “longevity regulating pathway”, and “tight junctions” [23,24], and another study showed that FTO could promote chicken adipogenesis through the Wnt signaling pathway [50], which further supports the possibility of a key role for ALKBH5 in muscle fiber type specification and another for FTO in adipogenesis via m^6^A demethylation. Zhang [49] demonstrated that the ALKBH5 protein is associated with fat deposition and proliferation in chickens, with higher expression in chicken abdominal fat compared to other adipose tissues, and knockdown of ALKBH5 increased the proliferation of chicken preadipocytes. In our current results, however, we did not find a significant role for FTO in chicken pectoral muscle tissue expression or myoblast differentiation. Nevertheless, some studies have emphasized FTO’s vital role in chicken liver fat metabolism, where it can be negatively regulated by miR-33 to decrease expression in chicken liver [25]. Additionally, ALKBH5 exhibited higher expression during differentiation compared to other family members over time, suggesting a pivotal role in the differentiation of chicken myoblasts. FTO also showed a tendency to be upregulated during temporal differentiation, but due to its expression in tissues, we hypothesized that it might then function during the embryonic period or early in the development of chickens. Further exploration into the role of ALKBH5 and FTO demethylation in avian species holds great significance. Such endeavors will undoubtedly contribute to a deeper understanding of the intricate regulatory mechanisms governed by m^6^A modifications.

## 5. Conclusions

In this study, bioinformatics identified seven chicken AlkB family genes. Compared to mammals, avian species maintained relative chromosomal order, with FTO least homologous and ALKBH5 most in birds. Protein–protein interaction analysis revealed potential interactions among ALKBH1, 2, 3, 5, 8, and FTO. The expression of AlkB genes exhibited an upward trend during myoblast differentiation, offering insights into their functions and evolution that could aid future research. Together, these findings underscore the concerted role of RNA demethylation in avian muscle development, provide a solid bioinformatic framework for the AlkB family as key research objects, and establish the primary cell system of gene knockout and overexpression. This study analyzed the genes’ function in muscle differentiation and growth, which will jointly accelerate research into their mechanisms and provide information that can be used for genetic or epigenetic intervention, so as to improve the quality and performance of poultry muscle.

## Figures and Tables

**Table 1:** Primer information.

Gene Name	Primer Sequences (5′ to 3′)	Annealing Temperature (°C)	Size (bp)
ALKBH1	F: TCGGCTCTTTCGCTTCTACC	60	91
	R: CGATCTGAACACCTGTCCCC		
ALKBH2	F: GGAGAGTGCTTGCTCCAAGA	60	238
	R: CCATCACTCGTCCACATGCT		
ALKBH3	F: AGTGGTGTGCTTTTGGGTGA	60	157
	R: AAGTAGGCCAGGCAAACTCC		
ALKBH4	F: TGGAGACGTTGTCAGGGAGA	60	187
	R: AGCTAAGCCCGTTGATGGAC		
ALKBH5	F: CCGGAGCCGAACCTTTGT	60	121
	R: CTCATGGCCGGCTCGC		
ALKBH8	F: AACATGAGTCTGCCGAGTGG	60	127
	R: CGGTGTTTGACGGGTTTTCC		
FTO	F: AATGGAGCTTATGACGAGCCT	60	199
	R: GAAGGGATGGCATTCTGGCT		
GAPDH	F: TCCTCCACCTTTGATGCG	60	146
	R: GTGCCTGGCTCACTCCTT		

**Table 2:** Characteristics analysis of AlkB family.

Gene Name	Protein ID	Protein Molecular Weight (kDa)	Number of Amino Acids	Isoelectric Point
ALKBH1	NP 001026723.1	41.39	371	8.36
ALKBH2	NP 001264426.2	28.61	247	9.24
ALKBH3	NP 001269306.2	32.83	286	6.28
ALKBH4	XP_046758526.1	35.59	317	6.48
ALKBH5	NP 001244130.1	43.35	374	9.02
ALKBH8	XP 004938883.2	84.46	746	8.56
FTO	NP 001172076.1	58.89	507	5.74

## Data Availability

The original data in the article can be obtained directly from the corresponding author upon reasonable request.
